# Metabolic Dysfunction-Associated Fatty Liver Disease and Bone Mineral Density in School-Aged Children in China: A Propensity Score-Matched Analysis

**DOI:** 10.3390/nu18040621

**Published:** 2026-02-13

**Authors:** Junting Liu, Hanyue Guo, Qin Liu, Tao Li, Guimin Huang, Dongqing Hou, Yijing Cheng, Fangfang Chen, Xinnan Zong, Shaoli Li

**Affiliations:** Capital Center for Children’s Health, Capital Medical University, Capital Institute of Pediatrics, Beijing 100020, China; 13834779139@163.com (H.G.); liuqin@mail.ccmu.edu.cn (Q.L.); socott@126.com (T.L.); guiminhuang@163.com (G.H.); dqhou@sina.com (D.H.); yijing_cheng@163.com (Y.C.); airechen@126.com (F.C.); xnzong@sina.com (X.Z.); shaolichina1@163.com (S.L.)

**Keywords:** children, obesity, metabolism, metabolic dysfunction–associated fatty liver disease, bone mineral density

## Abstract

**Background/Objectives**: To examine the association between metabolic dysfunction–associated fatty liver disease (MAFLD) and bone mineral density in school-aged children. To investigate the association between metabolic dysfunction-associated fatty liver disease (MAFLD) and bone mineral density among school-aged children using a propensity score-matched study design. **Methods**: A cross-sectional analysis was performed using baseline data from the Beijing Children and Adolescents Health Cohort, with samples collected between September 2022 and May 2023. The study included 5170 children aged 7–18 years. Standardized questionnaires collected behavioral, lifestyle, and dietary data. Anthropometric measurements (height, weight, waist circumference) were obtained to calculate body mass index (BMI). Fasting venous blood samples were analyzed for glucose and lipid profiles. Clinical assessments included pubertal development evaluation, abdominal ultrasound for hepatic steatosis, oscillometric blood pressure measurement, quantitative ultrasound for calcaneal bone mineral density (BMD), and bioelectrical impedance analysis for body fat percentage. MAFLD was diagnosed as hepatic steatosis combined with metabolic abnormalities (assessed via BMI, blood glucose, lipid levels, and blood pressure). Propensity score matching (PSM) was conducted at a 1:3 ratio between the MAFLD and non-MAFLD groups, matching on age, sex, and pubertal stage. Multiple linear regression, conditional logistic regression, and quantile regression (10th–90th percentiles) were used to analyze the association between MAFLD and BMD. **Results**: Of 5170 participants, 579 had MAFLD and were matched to 1737 non-MAFLD controls (standardized mean differences < 0.001). Children with MAFLD had higher BMI, body fat percentage, and waist circumference, and lower BMD versus controls. Multiple linear regression confirmed a significant negative association between MAFLD and BMD, which was stronger in boys and mid-pubertal children. Conditional logistic regression analyses further showed that boys with MAFLD had a higher risk of reduced BMD. The odds ratios were 1.77 (95% CI: 1.14–2.75) overall, 2.74 (95% CI: 1.56–4.81) among those aged 12–14 years, 1.81 (95% CI: 1.04–3.17) in mid-puberty, and 2.27 (95% CI: 1.17–4.40) in late puberty. Quantile regression revealed the strongest associations between MAFLD and BMD at the 40th–75th percentiles (regression coefficients: −9.5 to −6.7). **Conclusions**: MAFLD was associated with lower bone mineral density in children, with the strongest associations observed in the lower-to-middle range. Boys, children in mid-puberty, and those with obesity may represent particularly vulnerable groups with respect to bone health in the presence of MAFLD. This highlights the importance of early MAFLD identification and targeted interventions to mitigate long-term skeletal risks. Prospective studies are needed to clarify the causal pathways between MAFLD and pediatric bone health, and future research should integrate multiple factors to elucidate the underlying mechanisms.

## 1. Introduction

With the increasing prevalence of childhood obesity, ectopic fat accumulation has become more common, and hepatic steatosis is being detected with greater frequency through imaging modalities such as ultrasonography. Non-alcoholic fatty liver disease (NAFLD), which has traditionally been diagnosed in children based on imaging findings alone, does not adequately capture underlying metabolic abnormalities [1]. In contrast, metabolic dysfunction–associated fatty liver disease (MAFLD) is defined by hepatic steatosis accompanied by obesity or obesity-related metabolic disturbances, including dyslipidemia, hyperglycemia, or elevated blood pressure [2]. MAFLD is closely linked to obesity and metabolic dysregulation [3]. As the prevalence of childhood obesity continues to rise [4,5], MAFLD not only increases the risk of progressive liver disease but is also associated with multiple components of metabolic syndrome, such as insulin resistance and hypertension.

Bone mineral density (BMD) is a key indicator of bone health and is influenced by genetic, metabolic, and lifestyle factors. Approximately 90% of peak bone mass is achieved before the age of 20 [6], and BMD accrued during childhood and adolescence plays a critical role in determining bone health later in life.

In adult populations, accumulating evidence indicates a potential link between hepatic steatosis and bone health [7,8,9]. Some studies have reported higher BMD in individuals with MAFLD [10], whereas others have documented the opposite. Notably, two-sample Mendelian randomization analyses have not supported a causal relationship between MAFLD (or hepatic steatosis) and BMD [11]. These conflicting findings may be explained by several factors. For instance, some studies suggest that individuals with MAFLD and lower BMD tend to exhibit a higher prevalence of low body weight [12], whereas weight gain itself may positively affect BMD and mediate the association between hepatic steatosis and bone health [13,14,15]. Conversely, chronic inflammation, vitamin D deficiency, and sedentary lifestyles—factors commonly observed in individuals with MAFLD—may disrupt bone metabolism and adversely affect BMD [16]. Overall, the relationship between MAFLD and BMD in adults appears complex and influenced by multiple interacting factors.

In children and adolescents, existing evidence suggests that NAFLD or fatty liver disease is negatively associated with BMD [16,17]. Of particular relevance, modifiable dietary factors, such as excessive intake of added fructose and alcohol, play a central role in the development and prevention of MAFLD [18], underscoring the need to investigate how MAFLD—as a metabolic condition shaped by these influences—relates to other health outcomes, including bone health. Given that childhood and adolescence represent critical periods for bone-mass accrual, clarifying the relationship between MAFLD and BMD is essential for the early identification of potential skeletal health risks and the development of effective preventive strategies. However, studies specifically examining the association between MAFLD and BMD in pediatric populations remain scarce.

Therefore, utilizing population-based survey data and a propensity-score-matched design, the present study aimed to investigate the association between MAFLD and BMD in children. Our findings are intended to provide timely epidemiological evidence that can inform clinical awareness and guide future longitudinal research into the mechanisms and long-term skeletal consequences of MAFLD in children. Ultimately, elucidating this relationship is crucial for developing integrated strategies that protect both metabolic and skeletal health from an early age.

## 2. Materials and Methods

### 2.1. Study Population

The study population was drawn from the Beijing Children and Adolescents Health Cohort, a school-based cohort including students from kindergarten through high school. Participant recruitment was conducted using a stratified cluster sampling method within one administrative district of Beijing to ensure the representativeness of the target population in the region. The baseline survey was conducted in September 2022 and initially included 5741 participants. For the present analysis, cross-sectional data from 5170 primary and secondary school students were selected.

Eligible participants met the following inclusion criteria: (1) school-aged children aged 7–18 years; (2) completion of abdominal ultrasonography and calcaneal bone mineral density assessment; (3) availability of venous blood samples and biochemical measurements; and (4) completion of the questionnaire survey. Participants were excluded if they had missing basic anthropometric measurements (height, weight, or waist circumference) or had other acute or chronic diseases.

**Figure 1:**
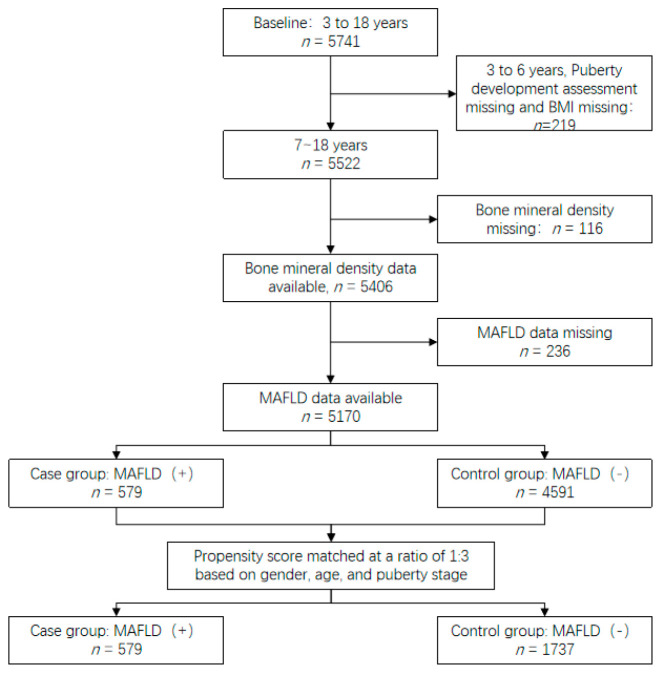
Flow chart of screening of study subjects.

Propensity score matching was conducted between children with MAFLD and those without MAFLD based on sex, age, and pubertal stage. An acceptable standardized mean difference of less than 0.1 was used to assess covariate balance. A 1:3 matching ratio was applied, resulting in 579 children in the MAFLD group and 1737 children in the control group, yielding a final matched sample of 2316 participants (Figure 1).

The study protocol was approved by the Ethics Committee of the Capital Institute of Pediatrics (SHERLL2022043). Written informed consent was obtained from the guardians of all participants and, where appropriate, from the participants themselves.

### 2.2. Research Methods

#### 2.2.1. Questionnaire Survey

A structured questionnaire was designed and developed by experienced physicians and researchers. This questionnaire was distributed only to participants who had signed the informed consent form, aiming to collect comprehensive information on demographic characteristics, screen time, sleep duration, moderate-to-vigorous physical activity (MVPA), and dietary habits. Specifically, information regarding diet, physical activity, and sleep was collected via self-report by the participants, and the dietary assessment adopted a food frequency questionnaire (FFQ) approach to retrospectively collect the frequency of dietary intake over a specified period. The questionnaire was completed jointly by students and their parents or guardians to ensure the accuracy and completeness of the reported information.

#### 2.2.2. Anthropometric Measurements

Anthropometric measurements were conducted exclusively for participants who had signed the informed consent form. Height was measured using standardized procedures and recorded to the nearest centimeter (cm). Body weight was assessed using a bioelectrical impedance body composition analyzer (H-key350, Beijing Sihai Huachen Technology Co., Ltd., Beijing, China) and recorded in kilograms (kg). Body mass index (BMI) was calculated as weight divided by height squared (kg/m^2^). Waist circumference was measured at the end of a normal expiration using a non-stretchable tape and recorded to the nearest centimeter.

#### 2.2.3. Clinical Examinations

Subsequent clinical examinations were also performed for the above-mentioned eligible participants (who had signed the informed consent form). Hepatic steatosis was assessed with a color Doppler ultrasound diagnostic system (LOGIQ V2, GE, Chicago, IL, USA) in the supine position, and images of the liver parenchyma were obtained. Bone mineral density (BMD) of the right calcaneus was measured using an ultrasonic bone densitometer (CM-200, Furuno, Nishinomiya, Japan) and expressed as speed of sound (SOS, m/s). Blood pressure was measured three times on the right upper arm using an oscillometric blood pressure monitor (HBP-1300, Omron, Kyoto, Japan). The average of the final two measurements was recorded as systolic blood pressure (SBP) and diastolic blood pressure (DBP), expressed in mmHg.

#### 2.2.4. Assessment of Pubertal Development

Pubertal development was assessed according to Tanner staging criteria [19]. In girls, breast development was evaluated by visual inspection and palpation, and pubic hair development was assessed visually; information on menarche status was also collected. In boys, pubic hair development and external genitalia were assessed visually, and testicular development was evaluated by palpation.

#### 2.2.5. Laboratory Measurements

Fasting venous blood samples were collected for biochemical analyses. Fasting plasma glucose (FPG) was measured using the hexokinase method. High-density lipoprotein cholesterol (HDL-C) was determined using the direct method, and triglycerides (TG) were measured using the glycerol phosphate oxidase–peroxidase (GPO-PAP) method. Alanine aminotransferase (ALT) levels were measured using the rate method. All biochemical assays were performed with Roche reagents. All biochemical parameters were expressed in mmol/L, except ALT, which was expressed in IU/L. Analyses were performed using fully automated biochemical analyzers (Siemens, Erlangen, Germany).

#### 2.2.6. Definitions and Grouping

Overweight and obesity were defined according to sex- and age-specific BMI cutoffs based on the Chinese standard Screening for Overweight and Obesity in School-aged Children and Adolescents [20]. Abdominal obesity was defined as a waist circumference at or above the 90th percentile for sex and age [21]. Hypertension was defined as SBP or DBP at or above the 90th percentile for sex and age, or a self-reported history of hypertension, in accordance with the Chinese Guidelines for the Prevention and Treatment of Hypertension in children [22]. Prediabetes was defined as an FPG level between 5.6 and 7.0 mmol/L, and diabetes was defined as an FPG level greater than 7.0 mmol/L or the use of glucose-lowering medication [23].

SOS values were categorized into quintiles, with the lowest quintile (20%) defined as low SOS. Fatty liver disease was diagnosed according to the Guidelines for the Diagnosis and Treatment of Non-alcoholic Fatty Liver Disease (2010 revision), based on increased liver parenchymal echogenicity relative to the kidneys on ultrasound examination [24]. MAFLD was diagnosed based on expert consensus on the management of lipid abnormalities in children and adolescents [25], which requires the presence of hepatic steatosis or elevated ALT levels, combined with specific metabolic abnormalities. The full diagnostic criteria are summarized in Table 1.

#### 2.2.7. Quality Control

All fieldwork procedures were strictly followed and aligned with the study protocol and investigation manual. Measurements were conducted by trained investigators and physicians. Anthropometric instruments, blood pressure monitors, bone densitometers, and body composition analyzers were calibrated daily to ensure measurement accuracy. Blood samples were transported under cold-chain conditions to the laboratory at the Capital Institute of Pediatrics within two hours of collection for processing and analysis.

#### 2.2.8. Data Processing and Statistical Analysis

Data were double-entered and cross-checked using EpiData version 3.0 to ensure accuracy. Continuous variables with a normal distribution were summarized as means with standard deviations, whereas non-normally distributed variables were described using medians and interquartile ranges. Categorical variables were presented as frequencies and percentages.

Multiple linear regression analyses were conducted to examine the association between MAFLD and calcaneal speed of sound (SOS), using both unadjusted and multivariable-adjusted models. The adjusted models included sex, age, pubertal stage, behavioral and lifestyle factors, and body mass index as covariates. Conditional logistic regression was applied to evaluate the association between MAFLD and the risk of low SOS, with results reported as odds ratios and 95% confidence intervals.

Forest plots were generated using the forestplot package in R. In addition, quantile regression analyses were performed to assess the association between MAFLD and SOS across the 10th to 90th percentiles, adjusting for relevant covariates. All statistical analyses were conducted in R (version 4.2.2), and a two-sided *p* value < 0.05 was considered statistically significant.

## 3. Results

### 3.1. Basic Characteristics Before and After Matching

Before propensity score matching, the proportion of males was higher in the MAFLD group than in the control group (72.4% vs. 48.7%). After matching, the proportions of males were comparable between the two groups (72.4% vs. 72.5%; SMD = 0.004). The mean age prior to matching was 11.8 years in the MAFLD group and 10.5 years in the control group; following matching, both groups had a mean age of 11.8 years (SMD = 0.020). Significant differences in pubertal stage distribution, particularly in the pre-pubertal and mid-pubertal stages, were observed before matching, but these differences were substantially attenuated after matching (SMD = 0.019). Overall, key baseline characteristics were well balanced between the two groups after propensity score matching.

Both before and after matching, children with MAFLD had higher body mass index, body fat percentage, and waist circumference, and a higher prevalence of reduced bone mineral density than controls (Table 2). Lifestyle and dietary characteristics before and after propensity-score matching are shown in Table 3.

### 3.2. Association Between MAFLD and Bone Mineral Density

Table 4 summarizes the results of multiple linear regression analyses examining the association between MAFLD and bone mineral density. In both the overall analysis and stratified analyses, MAFLD remained negatively associated with bone mineral density after adjustment for sex, age, pubertal stage, physical activity, screen time, dietary factors, and body mass index.

In the fully adjusted model, MAFLD was associated with lower bone mineral density, with a regression coefficient (β) of −5.90 (95% CI: −9.87 to −1.94). Among children aged 12–14 years, the association was stronger after full adjustment (β = −9.31, 95% CI: −15.71 to −2.91). Similarly, a significant negative association was observed in the mid-pubertal group (β = −7.28, 95% CI: −12.60 to −1.95). In boys, MAFLD was also significantly associated with lower bone mineral density after full adjustment (β = −5.28, 95% CI: −9.87 to −0.70).

In girls, the association between MAFLD and bone mineral density was not statistically significant in Models 1 and 2; however, after further adjustment for body mass index in Model 3, the association became significant (β = −9.10, 95% CI: −17.07 to −1.12).

### 3.3. Association Between MAFLD and Risk of Reduced Bone Mineral Density

**Figure 2:**
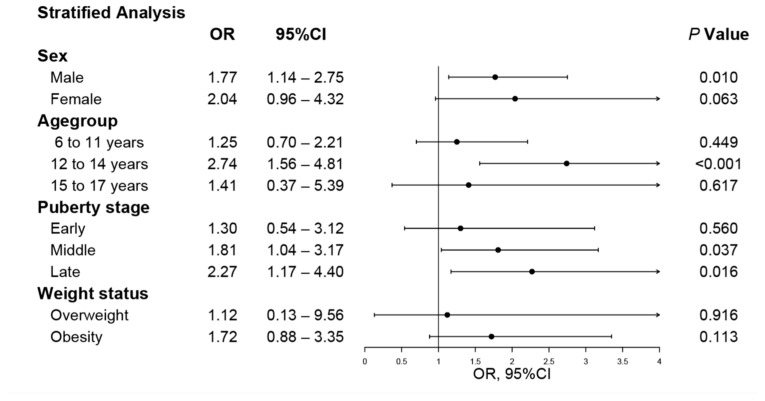
Effects of MAFLD on heel bone mineral density through logistic regression.

Figure 2 presents the results of stratified conditional logistic regression analyses examining the association between MAFLD and the risk of reduced bone mineral density, defined as BMD below the 20th percentile, after adjustment for all covariates. Among boys, MAFLD was significantly associated with an increased risk of reduced bone mineral density (OR = 1.77, 95% CI: 1.14–2.75; *p* = 0.010), whereas the association was not statistically significant among girls (*p* = 0.063).

In age-stratified analyses, the association between MAFLD and reduced bone mineral density was strongest among children aged 12–14 years (OR = 2.74, 95% CI: 1.56–4.81; *p* < 0.001). Stratification by pubertal stage showed significant associations in both mid-pubertal (OR = 1.81, 95% CI: 1.04–3.17) and late-pubertal participants (OR = 2.27, 95% CI: 1.17–4.40). In contrast, stratification by weight status indicated that the association between MAFLD and reduced bone mineral density was not statistically significant among overweight or obese children.

### 3.4. Effect of MAFLD on Bone Mineral Density Across Different Quantiles

Across quantiles, MAFLD was consistently associated with lower bone mineral density. Statistically significant associations (*p* < 0.05) were observed at the 20th, 25th, 30th, 40th, 50th, 60th, 70th, 75th, and 80th percentiles. The magnitude of the association varied across quantiles, with larger negative coefficients observed at the median and selected higher quantiles. Specifically, at the 50th percentile, the regression coefficient was −9.5 (*p* < 0.001), while coefficients of −6.5 and −8.0 were observed at the 40th and 75th percentiles, respectively (both *p* < 0.001).

Overall, the negative association between MAFLD and bone mineral density was evident across a broad range of the distribution, with more pronounced effects observed in the middle to higher quantiles (Table 5).

## 4. Discussion

### 4.1. MAFLD as a Risk Factor for Low Bone Mineral Density

Using a propensity score–matched design, the present study demonstrates that MAFLD is significantly associated with lower BMD and an increased risk of low BMD in a large pediatric population, particularly among boys and during mid-puberty. Our findings are consistent with previous pediatric studies. For instance, Lauren F. et al. reported a negative association between liver fat content and total body BMD in children aged 8–17 years using DXA and MRI [17]. Similarly, Labayen et al. found that hepatic steatosis was inversely related to BMD in overweight and obese children aged 8–12 years, independent of overall adiposity [26]. Other studies have shown that children with nonalcoholic steatohepatitis have lower BMD than those with simple steatosis [27,28], and elevated liver enzymes are also linked to reduced bone mass [17]. A meta-analysis by Sun et al. further indicated that children with both NAFLD and obesity are more likely to develop osteoporosis than those with obesity alone [29]. Collectively, these results support that MAFLD serves as an independent risk factor for impaired bone health in children, with stronger associations observed in specific subgroups such as males and mid-pubertal individuals.

### 4.2. Influence of Body Mass Index on the Association Between MAFLD and BMD

Body mass index appears to play a complex moderating role in the relationship between MAFLD and bone health. In our stratified analyses, no significant association was observed between MAFLD and BMD among overweight or obese children, suggesting that the protective mechanical loading effect of higher body weight may partly counteract the detrimental skeletal impact of hepatic steatosis. This observation aligns with findings from adult populations. For example, analyses of NHANES data revealed that the association between MAFLD and a lower risk of osteoporosis was attenuated after adjusting for BMI [14]. Two-sample Mendelian randomization studies also failed to establish a causal link between MAFLD and BMD [11]. Similarly, Zhang Wei et al. reported no independent association between MAFLD or liver fibrosis and BMD after accounting for obesity status [30]. Thus, BMI may act as an important effect modifier in the MAFLD–BMD relationship, particularly in individuals with higher body weight, where skeletal benefits from mechanical loading could mask the adverse effects of hepatic steatosis.

### 4.3. Potential Biological Mechanisms Linking MAFLD and Bone Health

Although the mechanisms underlying the association between MAFLD and reduced BMD are not fully understood, several biological pathways may be involved. Hepatic steatosis is commonly accompanied by chronic low-grade inflammation, vitamin D deficiency, and sedentary behavior, all of which may adversely affect bone metabolism [16]. Bone marrow adiposity represents a potential mechanistic link, as increased marrow fat has been positively correlated with hepatic steatosis in children and negatively associated with BMD [31,32,33,34]. Given the shared mesenchymal stem cell origin of osteoblasts and adipocytes, increased adipogenic differentiation within the bone marrow may occur at the expense of osteoblastogenesis [35].

In addition, inflammatory cytokines and adipokines are often elevated in children with obesity and MAFLD, potentially promoting bone resorption and impairing bone formation [36,37]. Adiponectin, an adipocyte-derived hormone, has been shown to influence bone remodeling through its receptors in both the liver and skeletal tissue, exerting complex effects on osteoblast and osteoclast activity [38]. Vitamin D deficiency, which is prevalent among children with obesity and metabolic disorders, may further compromise bone health by inducing apoptosis of bone-forming cells and disrupting calcium homeostasis [39]. Taken together, these mechanisms suggest that MAFLD may impair pediatric bone health through metabolic, inflammatory, and endocrine pathways.

### 4.4. Clinical and Public Health Implications

Given that approximately 90% of peak bone mass is accrued before adulthood, impaired bone development during childhood and adolescence may have long-term consequences for skeletal health. The present findings underscore the importance of considering bone health as a relevant comorbidity in children with MAFLD. Enhanced screening for low BMD in this population may be warranted, particularly among boys and those undergoing mid-to-late puberty.

Lifestyle interventions aimed at improving both metabolic and skeletal health may be beneficial. A balanced diet during childhood and adolescence is critical for lifelong bone health, with growing evidence supporting the positive impact of dietary patterns rich in fruits, vegetables, low-fat dairy products, whole grains, poultry, fish, nuts, and legumes on bone mineral accrual and maintenance [40]. In parallel, weight-bearing physical activity has been shown to increase lean mass and improve bone strength [41], while adequate dietary intake of calcium and vitamin D is essential for optimal bone mineralization. For children at particularly high risk of low bone mass, individualized interventions, including supplementation or pharmacologic therapy, may be considered with careful clinical judgment [42].

### 4.5. Strengths and Limitations

This study has several strengths, including its large population-based design and the use of propensity score matching to reduce confounding when examining the association between MAFLD and BMD. Detailed assessment of pubertal development allowed for more accurate adjustment for maturation-related changes in bone health. Nevertheless, several limitations should be acknowledged. First, BMD was assessed using quantitative ultrasound rather than DXA, which may introduce measurement variability. Quantitative ultrasound of the calcaneus may not fully capture bone health at other clinically relevant skeletal sites, such as the lumbar spine or femoral neck. Second, the cross-sectional design precludes causal inference, and longitudinal studies are needed to clarify temporal relationships. Third, hepatic steatosis was diagnosed by ultrasound without grading severity, which may have limited the precision of exposure classification. The lack of steatosis severity stratification prevents analysis of dose–response relationships between MAFLD severity and BMD reduction, which may obscure potential differences in skeletal risks across disease stages. Fourth, data on diet, sleep, and physical activity were collected via self-reported questionnaires, which are prone to recall bias. Specifically, such self-reported data cannot quantify absolute nutrient intake (e.g., the exact grams of added fructose or calcium consumed), which limits our ability to assess the direct impact of specific dietary components on the association between MAFLD and BMD.

## 5. Conclusions

In conclusion, MAFLD was independently associated with lower bone mineral density in children and adolescents, particularly among boys and during mid-to-late puberty. These findings highlight the need for increased awareness of bone health in pediatric patients with MAFLD and support further investigation into targeted prevention and intervention strategies for high-risk groups. For future research, prospective studies are warranted to elucidate the causal pathways linking MAFLD and bone health in this population. Given the multifactorial nature of MAFLD, subsequent research should integrate metabolic, hormonal, inflammatory, and behavioral factors to better characterize the mechanisms underlying skeletal impairment. Additionally, early identification of MAFLD combined with targeted interventions to support bone development may help mitigate the long-term risk of osteoporosis and fractures in children and adolescents.

## Figures and Tables

**Table 1:** Diagnostic Criteria for MAFLD.

Diagnostic Prerequisite (Satisfy Either)	Associated Metabolic Abnormality (Satisfy Any One)
1. Hepatic steatosis 2. Elevated ALT levels (≥2 times the upper limit of normal: ≥52 IU/L for males and ≥44 IU/L for females)	1. Overweight, obesity, or abdominal obesity 2. FPG ≥ 5.6 mmol/L 3. Normal body weight with one or more of the following: (1) abnormal triglyceride levels; (2) reduced HDL-C levels; (3) TG/HDL-C ratio > 2.25; (4) at least two metabolic abnormalities (blood pressure, TG, or HDL-C).

**Table 2:** Demographic, anthropometric, and metabolic characteristics before and after propensity-score matching.

Characteristic	Group	Before Propensity-Score Matching		SMD	After Propensity-Score Matching		SMD
		MAFLD (-)	MAFLD (+)		MAFLD (-)	MAFLD (+)	
no.		4591	579		1737	579	
Male sex—no. (%)		2234 (48.7)	419 (72.4) *	0.5	1260 (72.5)	419 (72.4)	0.004
Age, years		10.5 (3.1)	11.8 (2.6) *	0.483	11.8 (2.5)	11.8 (2.6)	0.02
Puberty Stage	Early	1895 (41.3)	111 (19.2) *	0.563	336 (19.3)	111 (19.2)	0.019
	Middle	1197 (26.1)	279 (48.2)		821 (47.3)	279 (48.2)	
	Late	1499 (32.7)	189 (32.6)		580 (33.4)	189 (32.6)	
BMI, kg/m^2^		19.1 (4.1)	28.2 (4.4) *	2.134	20.2 (4.1)	28.2 (4.4) *	1.877
FMP, %		22.5 (9.5)	37.1 (6.6) *	1.779	21.9 (9.5)	37.1 (6.6) *	1.857
WC, cm		65.0 (11.2)	90.7 (11.2) *	2.296	69.4 (11.5)	90.7 (11.2) *	1.883
Bone mineral density, m/s		1524.9 (29.9)	1520.1 (30.3) *	0.161	1525.4 (30.5)	1520.1 (30.3) *	0.175
Osteopenia		877 (19.1)	155 (26.8) *	0.183	326 (18.8)	155 (26.8) *	0.192
HDL-C		1.5 [1.3–1.7]	1.2 [1.0–1.3] *	−0.945	1.4 [1.2–1.7]	1.2 [1.0–1.3] *	−0.804
TG		0.8 [0.58–1.0]	1.2 [0.89–1.6] *	1.129	0.8 [0.6–1.1]	1.2 [0.9–1.6] *	0.948
FPG		4.9 [4.7–5.1]	5.0 [4.8–5.3] *	0.333	5.0 [4.8–5.2]	5.0 [4.8–5.3] *	0.145
ALT		11.6 [9.5–14.6]	26.7 [18.2–42.0] *	1.759	11.8 [9.6–15.2]	26.7 [18.2–42.0] *	1.201

* The difference is statistically significant compared to the control group.

**Table 3:** Lifestyle and dietary characteristics before and after propensity-score matching.

Characteristic	Group	Before Propensity-Score Matching		SMD	After Propensity-Score Matching		SMD
		MAFLD (-)	MAFLD (+)		MAFLD (-)	MAFLD (+)	
no.		4591	579		1737	579	
Screen time, minutes per day		60.0 [30.0, 120.0]	90.0 [60.0, 130.0] *	0.138	77.5 [40.0, 120.0]	90.0 [60.0, 130.0]	0.039
Moderate-to-vigorous physical activity time, minutes per week		100.0 [50.0, 160.0]	90.0 [42.0, 150.0]	0.066	105.0 [60.0, 200.0]	90.0 [42.0, 150.0] *	0.155
Sleeping time, hours per day		8.7 (1.0)	8.5 (1.0) *	0.178	8.5 (1.0)	8.5 (1.0)	0.005
Dietary frequency, ≥1 time per day							
Fish, Egg, Milk	No	1298 (29.6)	168 (30.4)	0.016	459 (27.8)	168 (30.4)	0.057
Vegetable	No	814 (18.4)	117 (21.0)	0.066	287 (17.2)	117 (21.0)	0.097
Meat	No	1745 (39.4)	224 (40.2)	0.016	621 (37.3)	224 (40.2)	0.061
Dietary frequency, ≥1 time per week							
Fry food	Yes	1840 (41.7)	211 (37.9)	0.078	708 (42.7)	211 (37.9)	0.096
Sugar-sweetened beverage	Yes	2138 (48.6)	280 (50.7)	0.042	896 (53.9)	280 (50.7)	0.064
Snack	Yes	3031 (68.6)	324 (58.6) *	0.21	1104 (66.2)	324 (58.6)	0.157
Drink in the past month	Tried drinking	142 (3.2)	19 (3.4)	0.038	67 (4.0)	19 (3.4)	0.044
	Have drunk	72 (1.6)	12 (2.1)		43 (2.6)	12 (2.1)	
Smoke in the past month	Tried smoking	9 (0.2)	3 (0.5)	0.054	5 (0.3)	3 (0.5)	0.085
	Smoked 1 cigarette	24 (0.5)	3 (0.5)		21 (1.3)	3 (0.5)	
Secondhand smoke in the past month	1 to 2 days	703 (16.1)	102 (18.2) *	0.124	272 (16.3)	102 (18.2)	0.109
	3 to 4 days	175 (4.0)	25 (4.5)		79 (4.7)	25 (4.5)	
	Almost every day	334 (7.6)	58 (10.4)		130 (7.8)	58 (10.4)	

* The difference is statistically significant compared to the control group.

**Table 4:** Effects of MAFLD on heel bone mineral density through multiple linear regression.

	Model 1			Model 2			Model 3		
	β (95%CI)	t	*p*	β (95%CI)	t	*p*	β (95%CI)	t	*p*
Overall model	−5.32 (−8.18, −2.45)	−3.64	<0.001	−4.77 (−7.75, −1.79)	−3.14	0.002	−5.90 (−9.87, −1.94)	−2.92	0.004
Stratified analysis									
Stratified by age group									
6 to 11 years	−3.33 (−7.06, 0.40)	−1.75	0.081	−3.34 (−7.24, 0.56)	−1.68	0.094	−4.47 (−9.67,0.73)	−1.68	0.093
12 to 14 years	−9.64 (−14.31, −4.96)	−4.04	<0.001	−7.60 (−12.43, −2.78)	−3.09	0.002	−9.31 (−15.71, −2.91)	−2.85	0.004
15 to 17 years	−0.47 (−9.35, 8.42)	−0.10	0.918	−0.97 (−11.32, 9.38)	−0.18	0.855	−5.85 (−19.90, 8.19)	−0.82	0.415
Stratified by sex									
Male	−7.45 (−10.80, −4.09)	−4.35	<0.001	−6.42 (−9.89, −2.94)	−3.62	<0.001	−5.28 (−9.87, −0.70)	−2.26	0.024
Female	0.26 (−5.19, 5.71)	0.09	0.926	−0.75 (−6.53, 5.03)	−0.26	0.799	−9.10 (−17.07, −1.12)	−2.24	0.026
Stratified by puberty stage									
Early	−4.46 (−10.00, 1.09)	−1.58	0.116	−5.25 (−11.25, 0.74)	−1.72	0.087	−6.32 (−14.39, 1.76)	−1.53	0.126
Middle	−9.34 (−13.24, −5.44)	−4.69	<0.001	−8.25 (−12.30, −4.21)	−4.00	<0.001	−7.28 (−12.60, −1.95)	−2.68	0.008
Late	0.14 (−5.42, 5.71)	0.05	0.960	0.60 (−5.35,6.56)	0.20	0.843	−5.19 (−13.35, 2.96)	−1.25	0.212
Stratified by BMI									
Overweight	−2.68 (−9.89, 4.54)	−0.73	0.468	−6.05 (−13.99, 1.89)	−1.49	0.136	-	-	-
Obesity	−4.15 (−8.43, 0.13)	−1.90	0.058	−3.99 (−8.44, 0.45)	−1.81	0.079	-	-	-

Note: Model 1 did not adjust for any confounders. Model 2 adjusted for sex, age, puberty stage, physical activity, screen time, dietary frequency, sleeping time, smoking, and drinking. The stratified factor was not adjusted. Model 3 further adjusted BMI.

**Table 5:** Effects of MAFLD on different percentiles of heel bone mineral density through quantile regression.

*τ*	β	Se	t	*p* Value
0.1	−4.6	2.4	−1.875	0.061
0.2	−5.2	2.1	−2.429	0.015
0.25	−4.6	2.3	−1.989	0.047
0.3	−6.4	2.0	−3.118	0.002
0.4	−8.4	1.9	−4.377	<0.001
0.5	−9.5	2.0	−4.735	<0.001
0.6	−7.7	2.5	−3.101	0.002
0.7	−8.0	2.6	−3.022	0.003
0.75	−7.7	2.8	−2.768	0.006
0.8	−6.7	3.1	−2.151	0.032
0.9	−5.4	4.2	−1.297	0.195

## Data Availability

The data are not publicly available due to privacy concerns. The raw data supporting the conclusions of this article will be made available upon request.
